# Circular PPP1R13B RNA Promotes Chicken Skeletal Muscle Satellite Cell Proliferation and Differentiation via Targeting miR-9-5p

**DOI:** 10.3390/ani11082396

**Published:** 2021-08-13

**Authors:** Xiaoxu Shen, Yuanhang Wei, Guishuang You, Wei Liu, Felix Kwame Amevor, Yao Zhang, Haorong He, Menggen Ma, Yun Zhang, Diyan Li, Qing Zhu, Huadong Yin

**Affiliations:** 1 Farm Animal Genetic Resources Exploration and Innovation Key Laboratory of Sichuan Province, Sichuan Agricultural University, Chengdu 611130, China; shenxiaoxu@stu.sicau.edu.cn (X.S.); weiyuanhang@stu.sicau.edu.cn (Y.W.); youguishuang@stu.sicau.edu.cn (G.Y.); 201804670@stu.sicau.edu.cn (W.L.); amevorfelix@gmail.com (F.K.A.); zhangyao@sicau.edu.cn (Y.Z.); hehaorong@stu.sicau.edu.cn (H.H.); diyanli@sicau.edu.cn (D.L.); 2 College of Resources, Sichuan Agricultural University, Chengdu 611130, China; mgen@sicau.edu.cn; 3 College of Management, Sichuan Agricultural University, Chengdu 611130, China; zhangyun@stu.sicau.edu.cn

**Keywords:** circPPP1R13B, miR-9-5p, SMSC, IGF2BP3, IGF/PI3K/AKT signaling pathway

## Abstract

**Simple Summary:**

The skeletal muscle of livestock is the main resource of proteins for human beings. Understanding the molecular mechanisms regulating the growth and development of skeletal muscle is important in breeding high yield quality muscle animals. In this study, we evaluated the expression of a novel circular RNA circPPP1R13B in fast muscle growing broilers and slow muscle growing layers, and the results showed that circular RNA circPPP1R13B was highly expressed in fast muscle growing broilers compared to the slow muscle growing layers. It was also revealed in this study that circPPP1R13B promote the proliferation and differentiation of chicken skeletal muscle satellite cells via targeting miR-9-5p. Therefore, the results obtained in this study indicate that circPPP1R13B may be a regulatory factor for promoting skeletal muscle growth and development in broilers, and possibly as a potential target for molecular breeding.

**Abstract:**

Skeletal muscle plays important roles in animal locomotion, metabolism, and meat production in farm animals. Current studies showed that non-coding RNAs, especially the circular RNA (circRNA) play an indispensable role in skeletal muscle development. Our previous study revealed that several differentially expressed circRNAs among fast muscle growing broilers (FMGB) and slow muscle growing layers (SMGL) may regulate muscle development in the chicken. In this study, a novel differentially expressed circPPP1R13B was identified. Molecular mechanism analysis indicated that circPPP1R13B targets miR-9-5p and negatively regulates the expression of miR-9-5p, which was previously reported to be an inhibitor of skeletal muscle development. In addition, circPPP1R13B positively regulated the expression of miR-9-5p target gene insulin like growth factor 2 mRNA binding protein 3 (IGF2BP3) and further activated the downstream insulin like growth factors (IGF)/phosphatidylinositol 3-kinase (PI3K)/AKT serine/threonine kinase (AKT) signaling pathway. The results also showed that the knockdown of circPPP1R13B inhibits chicken skeletal muscle satellite cells (SMSCs) proliferation and differentiation, and the overexpression of circPPP1R13B promotes the proliferation and differentiation of chicken SMSCs. Furthermore, the overexpression of circPPP1R13B could block the inhibitory effect of miR-9-5p on chicken SMSC proliferation and differentiation. In summary, our results suggested that circPPP1R13B promotes chicken SMSC proliferation and differentiation by targeting miR-9-5p and activating IGF/PI3K/AKT signaling pathway.

## 1. Introduction

Traditional breeding principles of domestic animals to attain economic gain take long periods to achieve the set-goals and objectives. Therefore, molecular breeding technology that could shorten breeding processes were used in the past decade [1,2]. The exploration of genes related to economic traits is the basis of molecular breeding technology, thus exploration of genes regulating important economic traits is regarded as a conducive scientific trait selection and also promotes the production performance of local domestic animals [3,4,5].

Skeletal muscle of domestic animals is the main protein source for humans as well as an important economic trait of domestic animals. The development of skeletal muscle is associated with the quality and quantity of animal meat [6], which is determined by the activity of myogenic cells, such as myoblasts and SMSCs [7]. The process of skeletal muscle development is precisely managed by several important transcription factors, including the myogenic regulatory factors (MRFs) and the myocyte enhancer factor 2 family (MEF2s) [8,9]. Furthermore, many non-coding RNAs have been shown to regulate muscle development in recent years.

CircRNA is a class of endogenous non-coding RNAs without 5′ and 3′ ends [10]. CircRNA was firstly discovered in Viroids in the early 1976, however, there is limited literature about due to its unique structure [11]. However, with the development of RNA sequencing technology, several circRNAs have been identified in various animal tissues and cells [12]. It is reported that circRNAs could act as miRNA sponges, to regulate miRNAs to alleviate its inhibitory effect on messenger RNAs (mRNAs) [13]. CDR1as promotes SMSCs differentiation by upregulating the expression of IGF1R via sponging miR-7 [14]. CircHIPK3 was reported to be involved in promoting C2C12 myoblast proliferation and differentiation via miR-7/transcription factor 12 (TCF12) axis [15]. CircARID1A promotes skeletal muscle regeneration via targeting miR-6368 [16]. In addition, circRILPL1 accelerates bovine myogenesis through sponging miR-145 [17]. Few circRNAs have been reported to regulate skeletal muscle development, however, molecular mechanisms of some potential circRNAs are not yet studied.

In chickens, broilers have fast muscle growth rate compared with the laying hens, this may be due to their physiological differences [18,19]. For instance, we previously sequenced circRNA in the breast muscle of a FMGB (ROSS 308) and a SMGL (White Leghorns) at different embryonic days (E10, E13, E16 and E19). We found that hundreds of differentially expressed circRNAs were identified, and among them a novel circRNA circTMTC1 was found to inhibit the differentiation of chicken SMSCs by up-regulating myostatin via sponging miR-128-3p [20]. In this study, we found that another novel circRNA (i.e., circPPP1R13B) was highly expressed in the FMGB compared with SMGL at all four time points (E10, E13, E16 and E19). Thus, in this study, we aimed to explore the potential functions of circPPP1R13B on chicken SMSCs proliferation, differentiation to reveal the underlying mechanisms.

## 2. Materials and Methods

### 2.1. Sample Collection

All animal experiments of this study were approved by the Animal Care Committee of Sichuan agricultural university with the approval number is 2020102012. A total of 200 Ross 308 broilers (1-day-old) used for cell culture were purchased from Sichuan Zhengda Food Co., Ltd. (Sichuan, China). For tissue expression pattern analysis, the skeletal muscle (breast muscle), fat, liver, spleen, heart, lung, brain and intestine tissues were collected from three chicks. The samples were rapidly frozen in liquid nitrogen and subsequently kept at −80 °C for further analysis.

### 2.2. siRNAs and Vectors

Small interfering RNAs (siRNAs) designed to overlap the junction site of circPPP1R13B and miRNA mimic were synthesized by GenePharma Co., Ltd. (Shanghai, China) (Supplementary Table S1). For circPPP1R13B overexpression, the whole linear sequence of circPPP1R13B was synthesized and inserted into pCD2.1-ciR (Geneseed Biotech, Guangzhou, China) according to the manufacturer’s instructions (ov-circPPP1R13B). The empty vector was set as a negative control (ov-NC). For dual-luciferase reporter assay, the wild type (WT) and mutant type (MT) of circPPP1R13B linear fragment which contains miR-9-5p binding site were synthesized and inserted into pmirGLO dual-luciferase reporter vector according to the manufacturer’s instructions. All constructed vectors were verified by sequencing.

### 2.3. Cell Culture and Treatment

Chicken SMSCs were isolated from the ROSS-308 chicken breast muscle (at day 4) as described in our previous study [21]. SMSCs were cultured in the growth medium [Dulbecco’s modified Eagle’s medium (DMEM) (Gibco, Langley, OK, USA) + 10% fetal bovine serum (Gibco, Langley, OK, USA)]. When SMSCs were induced to differentiate, the medium was replaced with differentiation medium [DMEM + 2% horse serum (Gibco, Langley, OK, USA)]. Besides, DF-1 cells (Fuheng biology, Shanghai, China) were cultured in growth medium. All medium was changed every 24 h.

SMSCs were transfected by Lipofectamine 3000 kit (Invitrogen, Carlsbad, CA, USA) according to the manufacturer’s instructions. For circPPP1R13B functional analysis, transfection was set to five groups, si-PPP1R13B, siRNA-NC, co-transfected ov-NC and mimic NC, co-transfected ov-NC and miR-9-5p mimic, and co-transfected with ov-circPPP1R13B and miR-9-5p mimic. The SMSCs were transfected at approximately 90% confluence to study SMSCs differentiation. When cell proliferation was studied, the cells were transfected at 50% confluence.

### 2.4. Quantitative Real-Time PCR (qRT-PCR)

Total RNA from tissues and SMSCs was separated using RNAiso plus reagent (Takara, Tokyo, Japan). Reverse transcription reaction was performed using TransScript One-Step gDNA Removal and cDNA Synthesis SuperMix kit (for circRNA and mRNA; TransGen, Beijing, China) and One Step miRNA cDNA Synthesis Kit (for miRNA; HaiGene, Haerbin, China) according to the manufacturer’s instructions. qRT-PCR assay was performed in a final volume of 10 μL reaction, which contained 5μL TB Green PCR Master Mix (Takara, Tokyo, Japan), 0.5 μL forward primer, 0.5 μL reverse primer and 3μL DNase and RNase-free water. The samples were detected by a CFX Connect Real-Time System and three independent repetitions were performed for each sample. The primers were designed using Primer 5.0 software and listed in Supplementary Table S2. β-actin (for circRNA and mRNA) and U6 snRNA (for miRNA) were used as endogenous controls. The 2^−ΔΔCt^ method was used to analyze the qRT-PCR data. For circPPP1R13B identification, circPPP1R13B was amplified using qRT-PCR primer in the cDNA of the chicken muscle samples, the amplification product was sequenced by Sanger sequencing. RNase R was used for circRNA verification, because RNase R degrades the linear RNA but not circular RNA. We incubated 1μg mixed total RNAs of the chicken’s muscle tissues with 1-unit RNase R (Epicentre Technologies, Madison, WI, USA) at 37 °C for 10 min. Furthermore, RNase R was inactivated at 95 °C for 10 min, then the cDNAs were generated according to the general reverse transcription procedures. Thereafter, the reverse transcription assay with different primers were used for circRNA identification, because oligo d(T) primers cannot efficiently reverse transcribe circRNAs without poly (A) tail. The 2 μg mixed total RNAs of the chicken muscle tissues were divided into half, and one half was reverse transcribed with oligo d(T) primers, whereas the other half was reverse transcribed with Random N9 primers.

### 2.5. Western Blot

Western blot assay was performed to detect the protein levels of myogenic differentiation marker gene and the key proteins of IGF/PI3K/AKT signaling pathway, as previously described [22]. The primary antibodies were used are anti-myogenin (MyoG) (Biorbyt, Cambridge, UK; dilute 1:1000), anti-IGF2BP3 (ABclonal, Wuhan, China; 1:1000), anti-AKT1 (Cell Signaling Technology, Boston, MA, USA; 1:1000), anti-p-AKT1 (Cell Signaling Technology, Boston, MA, USA; 1:1000), anti-Glyceraldehyde-3-phosphate dehydrogenase (GAPDH) (ZenBio, Chengdu, China; 1:2000), and anti-β-tubulin (ZenBio, Chengdu, China; 1:2000). GAPDH and β-tubulin were used as endogenous controls. The relative protein levels were measured using Image J software.

### 2.6. Dual-Luciferase Assay

Dual-luciferase assay was performed to detect the combination between circPPP1R13B and miR-9-5p. Hence, the DF-1 cells were plated into a 48-well plate and co-transfected miR-9-5p mimic + WT luciferase reporter vector, miR-9-5p mimic + MT vector, negative mimic + WT vector and negative mimic + MT vector, respectively, and each treatment had three independent replicates. After 48 h transfection, cells were lysed and the relative luciferase activities were detected using Luc-Pair™ Duo-Luciferase HT Assay Kit (GeneCopoeia, Rockville, MD, USA) according to the manufacturer’s instructions on a fluorescence/multi-detection microplate reader (US BioTek Laboratories, Shoreline, WA, USA).

### 2.7. 5-Ethynyl-2′-Deoxyuridine (EdU) Assays

EdU assay was performed to detect the proliferation of SMSCs. Hence, the SMSCs were plated into a 96-well plate and transfected as described in the “2.3. Cell culture and treatment” section of this study. Each treatment had three independent replicates. After transfection for 48 h, the cells were incubated at 37 °C with 50 μm EdU for 2 h, then the EdU positive cells were measured using a Cell-Light EdU Apollo567 In Vitro Kits (RiboBio, Guangzhou, China) according to the manufacturer’s instructions. Three randomly selected fields were captured using a fluorescence microscope (Olympus, Tokyo, Japan), and the proliferating cells were stained by EdU, whereas the nuclei of the cells were stained by Hoechst 33342. The number of EdU positive cells and total cells was measured using Image-Pro Plus software.

### 2.8. Flow Cytometry Analysis

Flow cytometry analysis was performed to analyze the cell cycle of the SMSCs. Hence, the SMSCs were plated into a 12-well plate and transfected as described in the “2.3. Cell culture and treatment” section of this study. Each treatment had three independent replicates. After transfection for 48 h, then the cells were collected and digested to obtain cell suspension, which was resuspended in 75% precooled ethanol and fixed for 24 h. Thereafter, the cells were incubated with 500 μL PI/RNase Staining Buffer Solution (BD Biosciences, Franklin Lakes, NJ, USA) at 37 °C for 15 min, then BD AccuriC6 flow cytometer (BD Biosciences, Franklin Lakes, NJ, USA) was used for the Flow cytometric analysis.

### 2.9. Immunofluorescence

The immunofluorescence assay of the myosin heavy chain (MyHC) protein was performed to analyze the myotube formation of the SMSCs. Therefore, SMSCs were plated into a 24-well plate and transfected as described in “2.3. Cell culture and treatment” section of this study. Each treatment had three replicates. After the transfection and induced differentiation of the SMSCs for 72 h, the cells were fixed in a 4% formaldehyde for 30 min and permeabilized by 0.1% Triton X-100 for 20 min and then blocked by 5% goat serum (Beyotime, Shanghai, China) at room temperature for 30 min. Thereafter, the cells were incubated with the antibody anti-MyHC (Santa Cruz Bio, Santa cruz, CA, USA; 1:250) at 4 °C for 12 h, and then incubated with the Rhodamine (TRITC) AffiniPure Goat Anti-Mouse IgG (ZenBio, Chengdu, China; 1:1000) at 37 °C for 1 h. Finally, the cell nuclei were stained using DAPI (Beyotime, Shanghai, China; 1:50) for 5 min. Thereafter, three images were randomly captured using a fluorescence microscope (Olympus, Tokyo, Japan). The relative myotube area was measured using Image-Pro Plus software.

### 2.10. Statistical Analysis

Data are presented as least squares means ± standard error of the mean (SEM) and statistical analysis was performed using SPSS 20.0 software (IBM, Armonk, NY, USA). The qualified data after homogeneity test for variance was used for subsequent analysis. The significant difference between means were determined based on the unpaired Student’s t-test for two comparison groups, and one-way ANOVA for multiple comparison groups. Furthermore, the Turkey HSD method was used for the multiple comparison test of one-way ANOVA. *p* < 0.05 was considered to indicate statistical significance.

## 3. Results

### 3.1. Circular PPP1R13B RNA Is Differentially Expressed between FMGB and SMGL

**Figure 1:**
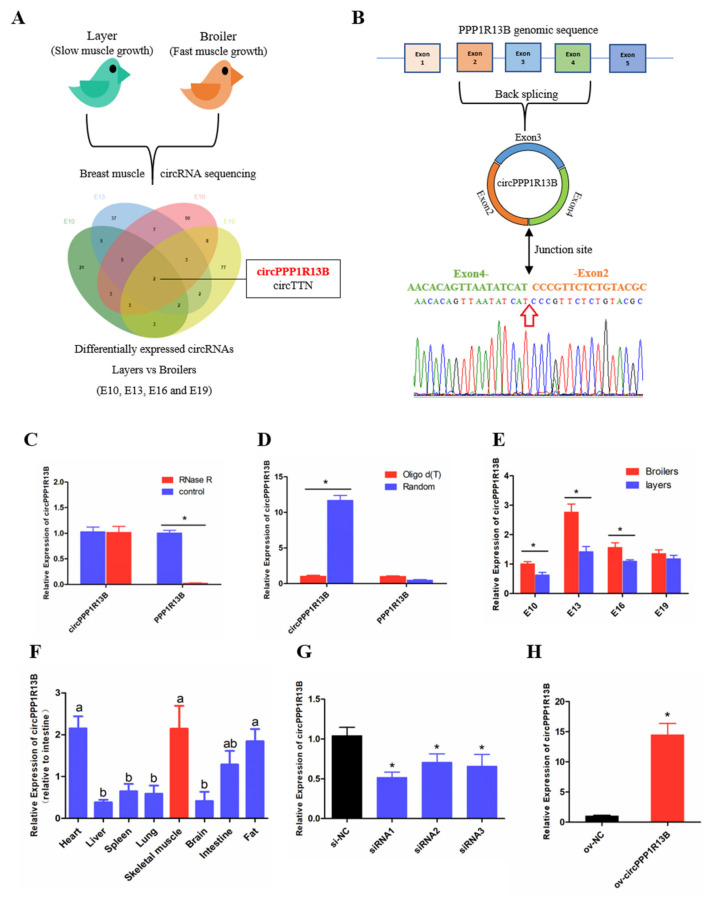
The selection, identification and expression pattern of circPPP1R13B. (**A**) differentially expressed circRNA analysis revealed that circPPP1R13B was chosen as a candidate circRNA, (**B**) The formation diagram and junction sequence of circPPP1R13B. (**C**) The expression of circPPP1R13B and linear PPP1R13B under RNase R treatment. (**D**) The expression of circPPP1R13B and linear PPP1R13B in reverse transcription samples with different primers. (**E**) The expression of circPPP1R13B in breast muscles of broilers and layers at four embryonic stage. (**F**) The expression of circPPP1R13B in different tissues of 4-day-old chickens. (**G**) Knock down efficiency of circPPP1R13B in SMSCs by exogenous siRNAs. (**H**) Overexpression efficiency of circPPP1R13B in SMSCs by overexpress vectors. Asterisk and different letters indicate significant differences from the respective control (* *p* < 0.05; ^a,b^ *p* < 0.05).

From our previous sequencing data (in the Sequence Read Archive database with accession number PRJNA516545), we found that circPPP1R13B was one of the differentially expressed circRNAs among FMGB and SMGL at all four time points (E10, E13, E16 and E19) (Figure 1A). Furthermore, we identified that circPPP1R13B came from the exon 2—4 of PPP1R13B gene, and the junction sequence which connects the head of exon 2 and the tail of exon 4 (Figure 1B). Our results also showed that PPP1R13B mRNA was digested by RNase R instead of circPPP1R13B (*p* < 0.05; Figure 1C), and in the reverse transcription reaction, random primers promoted circPPP1R13B cDNA synthesis more than oligo d (T) (*p* < 0.05; Figure 1D). These results confirmed the circular form of circPPP1R13B. Thereafter, the expression of circPPP1R13B in the chicken was identified by qRT-PCR. The results were similar to the sequencing data, which showed that the expression of circPPP1R13B was higher in the FMGB as compared to that in the SMGL at E10, E13 and E16 (*p* < 0.05; Figure 1E). Furthermore, we found that circPPP1R13B was also expressed in the fat, liver, spleen, heart, lung, brain, and intestine tissues, however, its expression was higher in skeletal muscle, heart and fat tissues than the other tissues (*p* < 0.05; Figure 1F).

In order to explore the potential function of circPPP1R13B in chicken skeletal muscle development, siRNAs and overexpression vector were used to control the expression of circPPP1R13B. The results showed that siRNAs, especially siRNA1 could significantly reduce the expression of circPPP1R13B, therefore siRNA1 was chosen for further experiments (named si-circPPP1R13B) (*p* < 0.05; Figure 1G). Besides, overexpression vector significantly increased the expression of circPPP1R13B (*p* < 0.05; Figure 1H).

### 3.2. CircPPP1R13B Interacts with miR-9-5p in Chicken SMSCs

**Figure 2:**
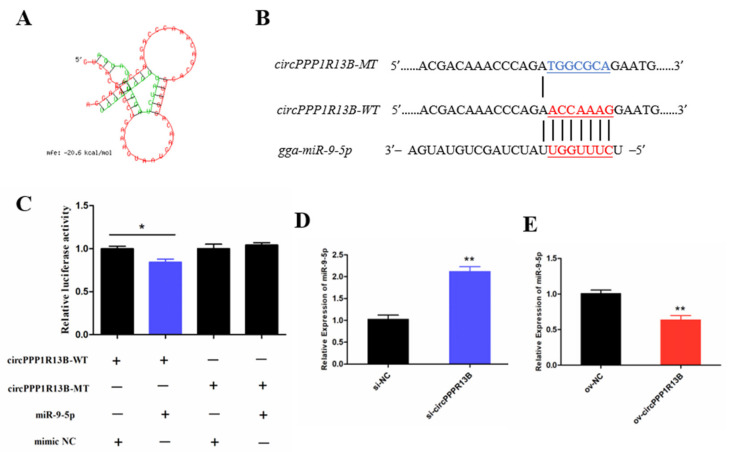
CircPPP1R13B directly targets miR-9-5p. (**A**) the target site of miR-9-5p on circPPP1R13B predicted by RNAhybrid software. (**B**) wild type and mutant type of miR-9-5p targeting site used for dual luciferase reporter vector construction. (**C**) Relative luciferase activity of wild type or mutant type reporter vectors co-transfected with miR-9-5p mimic or negative mimic. (**D**) The expression of miR-9-5p after circPPP1R13B knockdown in chicken SMSCs. (**E**) The expression of miR-9-5p after circPPP1R13B overexpression in chicken SMSCs. Asterisk indicate significant differences from the respective control (*^,^** *p* < 0.05).

Previous studies indicated that a circRNA functions as a miRNA sponge [23]. To verify whether circPPP1R13B sponges the miRNAs, RNAhybrid software was used for miRNA target prediction. The results showed that eighteen miRNAs directly target circPPP1R13B (Supplementary Table S3), among them, miR-9-5p was our focus (Figure 2A) due to the finding of our previous study which reported that, miR-9-5p could regulate proliferation and differentiation in chicken SMSCs [24]. To observe the relation between circPPP1R13B and miR-9-5p, dual luciferase reporter vector which contains wild type or mutant type binding site of miR-9-5p were constructed (Figure 2B), and co-transfected with miR-9-5p or negative mimic into the DF-1 cells. The results showed that miR-9-5p significantly reduced the luciferase of 3′ UTR which contain miR-9-5p wild type binding site, but had no effect on the luciferase with mutant type binding site (*p* < 0.05; Figure 2C). In addition, knockdown of circPPP1R13B significantly elevated miR-9-5p RNA level (*p* < 0.05; Figure 2D), whereas overexpression of circPPP1R13B significantly decreased the expression of miR-9-5p (*p* < 0.05; Figure 2E). These results suggested that circPPP1R13B acts as a sponge of miR-9-5p.

### 3.3. CircPPP1R13B Regulates IGF2BP3 via Targeting miR-9-5p in Chicken SMSCs

**Figure 3:**
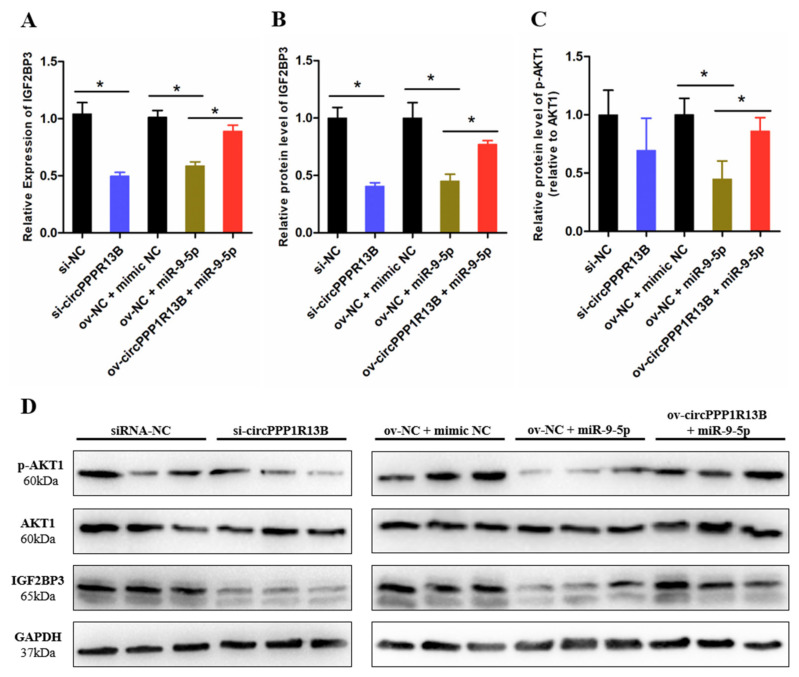
CircPPP1R13B regulates IGF2BP3 mediated IGF/PI3K/AKT signaling via targeting miR-9-5p. (**A**) Relative mRNA expression of IGF2BP3 following circPPP1R13B knockdown or co-overexpression with miR-9-5p. (**B**) Relative protein level of IGF2BP3 following circPPP1R13B knockdown or co-overexpression with miR-9-5p. (**C**) Relative protein phosphorylation level of AKT1 following circPPP1R13B knockdown or co-overexpression with miR-9-5p. (**D**) Protein levels of IGF2BP3, *p*-AKT1, AKT1, and GAPDH were determined by western blot analysis. Asterisk indicate significant differences from the respective control (* *p* < 0.05).

IGF2BP3 is a direct target gene of miR-9-5p, miR-9-5p inhibits the expression levels of IGF2BP3 and blocks the activity of downstream IGF/PI3K/AKT signaling pathway [24]. To verify whether circPPP1R13B regulates the expression of IGF2BP3 and downstream signaling pathway via targeting miR-9-5p, we performed co-transfection experiment, and the results showed that, the knockdown of circPPP1R13B decreased the RNA level of IGF2BP3, whereas the overexpression of miR-9-5p inhibited the mRNA expression of IGF2BP3 and co-overexpression of both miR-9-5p and circPPP1R13B restored the RNA level of IGF2BP3 (*p* < 0.05; Figure 3A). The protein level of IGF2BP3 also showed similar trend as the RNA level results (*p* < 0.05; Figure 3B–D). Furthermore, the key regulator of IGF/PI3K/AKT signaling pathway (phosphorylation of AKT1), the results showed the same trend after co-transfection (*p* < 0.05; Figure 3C,D).

### 3.4. CircPPP1R13B Eliminates the Inhibition Effect of miR-9-5p on Chicken SMSC Proliferation

**Figure 4:**
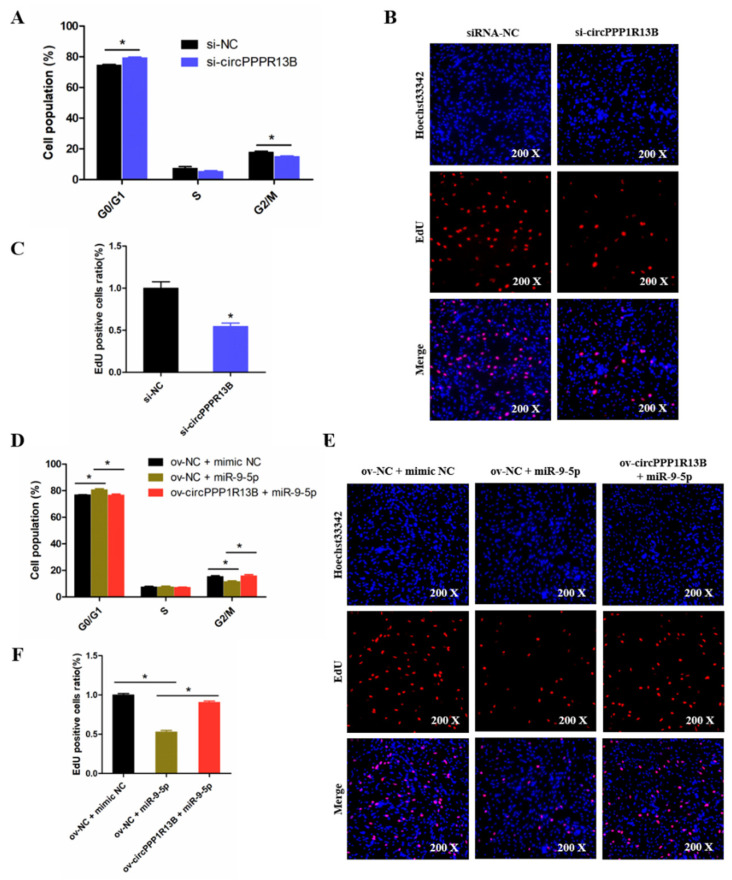
CircPPP1R13B regulates chicken SMSCs proliferation by targeting miR-9-5p. (**A**) Cell cycle analysis of SMSCs following circPPP1R13B knockdown. (**B**) EdU analysis plot of SMSCs following circPPP1R13B knockdown. (**C**) Relative EdU positive cells following circPPP1R13B knockdown. (**D**) Cell cycle analysis of SMSCs following co-overexpress circPPP1R13B with miR-9-5p. (**E**) EdU analysis plot of SMSCs following co-overexpress circPPP1R13B with miR-9-5p. (**F**) Relative EdU positive cells following co-overexpress circPPP1R13B with miR-9-5p. Asterisk indicate significant differences from the respective control (* *p* < 0.05).

To explore the role of circPPP1R13B in chicken SMSC proliferation, cell cycle analysis and EdU assay were performed in the circPPP1R13B knockdown SMSCs. Cell cycle analysis results showed that the knockdown of circPPP1R13B makes the SMSCs stagnant during the G0/G1 phase, and reduce the number of cells in division (G2/M) phase (*p* < 0.05; Figure 4A). EdU assay results showed that, the knockdown of circPPP1R13B decreased the number of EdU labeled proliferating cells (*p* < 0.05; Figure 4B,C). These results indicated that circPPP1R13B may play positive roles in SMSC proliferation.

Furthermore, we explored the relation between circPPP1R13B and miR-9-5p in SMSC proliferation. Cell cycle analysis results showed that, the overexpression of miR-9-5p reduced the population of G2/M phase cells and increased the population of G0/G1 phase cells, whereas the effect of miR-9-5p on SMSC cell cycle was alleviated by co-overexpressed miR-9-5p and circPPP1R13B (*p* < 0.05; Figure 4D). Similar to cell cycle analysis results, the EdU assay showed that the overexpression of miR-9-5p decreased the number of EdU positive cells, whereas the inhibition role of miR-9-5p on cell proliferation was eliminated by the co-overexpression with circPPP1R13B (*p* < 0.05; Figure 4E,F). Thus, these results suggested that circPPP1R13B eliminated the inhibition effect of miR-9-5p on chicken SMSC proliferation.

### 3.5. CircPPP1R13B Attenuates the Inhibition Effect of miR-9-5p on Chicken SMSC Differentiation

**Figure 5:**
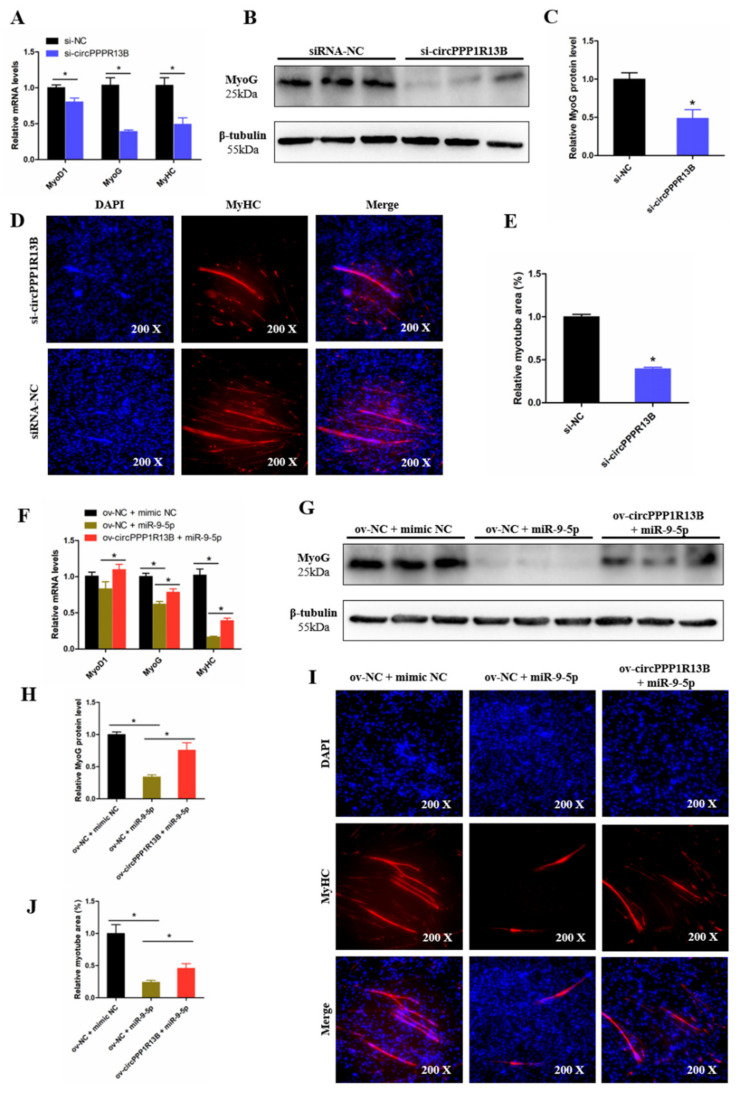
CircPPP1R13B regulates chicken SMSCs differentiation by targeting miR-9-5p. (**A**) Relative expression of muscle cell differentiation marker genes following circPPP1R13B knockdown. (**B**) Protein level of MyoG and β-tubulin observed by western blot analysis. (**C**) Relative protein level of MyoG following circPPP1R13B knockdown. (**D**) Images of MyHC staining of SMSCs following circPPP1R13B knockdown. (**E**) Relative myotube area of SMSCs following circPPP1R13B knockdown. (**F**) Relative expression of muscle cell differentiation marker genes following circPPP1R13B co-overexpress circPPP1R13B with miR-9-5p. (**G**) Protein level of MyoG and β-tubulin observed by western blot analysis. (**H**) Relative protein level of MyoG following circPPP1R13B co-overexpress circPPP1R13B with miR-9-5p. (**I**) Images of MyHC staining of SMSCs following circPPP1R13B co-overexpress circPPP1R13B with miR-9-5p. (**J**) Relative myotube area of SMSCs following circPPP1R13B co-overexpress circPPP1R13B with miR-9-5p. Asterisk indicate significant differences from the respective control (* *p* < 0.05).

To observe the function of circPPP1R13B on chicken SMSC differentiation, qRT-PCR, Western blot, and Immunofluorescence were performed in the circPPP1R13B knockdown SMSCs. qRT-PCR results showed that, the knockdown of circPPP1R13B significantly inhibited the RNA level of skeletal muscle development marker genes MyoG, Myogenic Differentiation 1 (MyoD1) and MyHC (*p* < 0.05; Figure 5A), Western blot result was similar to qRT-PCR data, which showed the protein level of MyoG were decreased by circPPP1R13B knockdown (*p* < 0.05; Figure 5B,C). In addition, the knockdown of circPPP1R13B inhibits the level of myotube component protein MyHC, which marks the formation of myotubes (*p* < 0.05; Figure 5D,E). These results showed that circPPP1R13B may play a positive role in chicken SMSC differentiation.

Furthermore, we explored the relation between circPPP1R13B and miR-9-5p on SMSC differentiation, and we observed that, the overexpression of miR-9-5p decreased the RNA level and protein level of skeletal muscle development marker genes, whereas the inhibitory effect of miR-9-5p on SMSC differentiation was prevented after the co-transfection with circPPP1R13B (*p* < 0.05; Figure 5F–H). Furthermore, miR-9-5p inhibited myotube formation, co-overexpression of the circPPP1R13B and miR-9-5p prevented the inhibition of miR-9-5p on myotube formation (*p* < 0.05; Figure 5I, J). Altogether, these results suggested circPPP1R13B attenuated the inhibition effect of miR-9-5p on chicken SMSC differentiation.

## 4. Discussion

The meat of farm animals such as pig, beef cattle, goat, fish and poultry serves as a major source of protein for humans. Understanding the mechanisms regulating the development of skeletal muscle is valuable to improve meat yield in farm animals. In the chicken, researches have shown that there are obvious phenotypic differences between FMGB and SMGL [4,18]. The underlying mechanism involves series of biological events, which contains many genes, signaling molecules and non-coding RNAs [20,25,26].

CircRNA is a novel class of non-coding RNAs, whose impact on the molecular mechanism of cellular machinery has not been fully established. Current studies have showed that circRNAs are involved in the chicken skeletal muscle development [27]. For instance, circSVIL promotes chicken myoblasts growth and differentiation via sponging miR-203 [28]. circFGFR2 was also found to boost both the proliferation and differentiation of the chicken myoblasts by binding miR-133a-5p and miR-29b-1-5p [29]. In the present study, we re-scanned the differentially expressed circRNAs between FMGB and SMGL, and we found that circPPP1R13B was presence in all the four groups (E10, E13, E16 and E19), this indicates that it plays a potential role in skeletal muscle development of chickens. In the SMSCs, the cell cycle analysis showed that circPPP1R13B could significantly promote the processes involved in cell cycling in order to promote cell division and multiplication. In addition, the results obtained from EdU analysis also indicated that circPPP1R13B could increase the number of EdU positive proliferation of SMSCs. These results revealed that circPPP1R13B promotes SMSC proliferation. Furthermore, myogenic differentiation is important for muscle development, which is regulated by the MRFs including MyoD1 and MyoG [9]. Our results showed that circPPP1R13B could increase the mRNA expression of MyoD1 and MyoG, and the protein level of MyoG. In addition, MyHC is an integral part of myotubes, and we found that circPPP1R13B significantly increased the relative area of MyHC labeled myotubes. Thus, our data suggested that circPPP1R13B could promote SMSC differentiation.

Studies shown that circRNAs could act as a miRNA sponge to release the inhibition effect of miRNAs on their targeted genes [30]. To investigate the role of circPPP1R13B in chicken myogenesis, target miRNAs of circPPP1R13B were predicted. Among those miRNAs, our focus was on miR-9-5p, because in our previous study, we found that miR-9-5p was highly expressed in the SMGL than FMGB, as well as inhibits chicken SMSC proliferation and differentiation via the IGF/PI3K/AKT signaling pathway [24]. Furthermore, we discovered that circPPP1R13B displayed an opposite expression pattern compared with miR-9-5p, hence, we conclude that circPPP1R13B may functions by targeting miR-9-5p. Therefore, we performed qRT-PCR analysis and dual-luciferase assay to confirmed that circPPP1R13B could inhibit the expression and function of miR-9-5p through miRNA response element. Furthermore, through the qRT-PCR analysis and western blot assay, we found that circPPP1R13B up-regulated the mRNA and protein expressions of IGF2BP3 via inhibiting miR-9-5p. Hence, our study indicated that circPPP1R13B could regulate the expression of IGF2BP3 by targeting miR-9-5p.

IGF2BP3, a regulator of chicken skeletal muscle development, had a higher expression level in a normal growing chicken than a chicken with a stunted growth [31], and promotes the proliferation and differentiation of chicken SMSCs through IGF/PI3K/AKT pathway [24]. IGF/PI3K/AKT signaling pathway facilitates proliferation and differentiation of skeletal muscle cells [32]. Reports indicated that IGF/PI3K/AKT signaling regulated by many non-coding RNAs in the skeletal muscle cells development. For instance, lncRNA lncIRS1 prevents skeletal muscle atrophy by targeting miR-15 family to active IRS1/PI3K/AKT signaling [33]. circRNA circTTN facilitates myoblast proliferation and differentiation via miR-432/IGF2/PI3K/AKT signaling axis [34]. In this study, we identified the relationship between circPPP1R13B and miR-9-5p/IGF2BP3 axis. Further, to verify whether circPPP1R13B could regulate the downstream IGF/PI3K/AKT signaling via targeting miR-9-5p, we performed co-overexpression experiments, and the results revealed that the inhibition effect of miR-9-5p on IGF/PI3K/AKT signaling were blocked with overexpression of both miR-9-5p and circPPP1R13B simultaneously. Thus, our results confirmed our hypothesis that circPPP1R13B regulates the IGF/PI3K/AKT signaling by targeting miR-9-5p, which further accelerates the proliferation and differentiation of chicken SMSCs. Besides, our study also found a novel upstream regulator circPPP1R13B of miR-9-5p and IGF/PI3K/AKT signaling.

Due to the exploration of functional genes of animals, molecular breeding technology has made great progress in animal breeding. Myostatin (MSTN) gene is a famous inhibitor of skeletal muscle development, the genomic mutation leads to the dysfunction of MSTN, hence, can be used to improve meat performance in domestic animals [2,3]. circPPP1R13B plays promotion role in the development of chicken skeletal muscle, therefore, the genomic mutation which caused the high expression of circPPP1R13B could possibly be a potential target for chicken molecular breeding to improve meat production in meat-type chickens.

## 5. Conclusions

**Figure 6:**
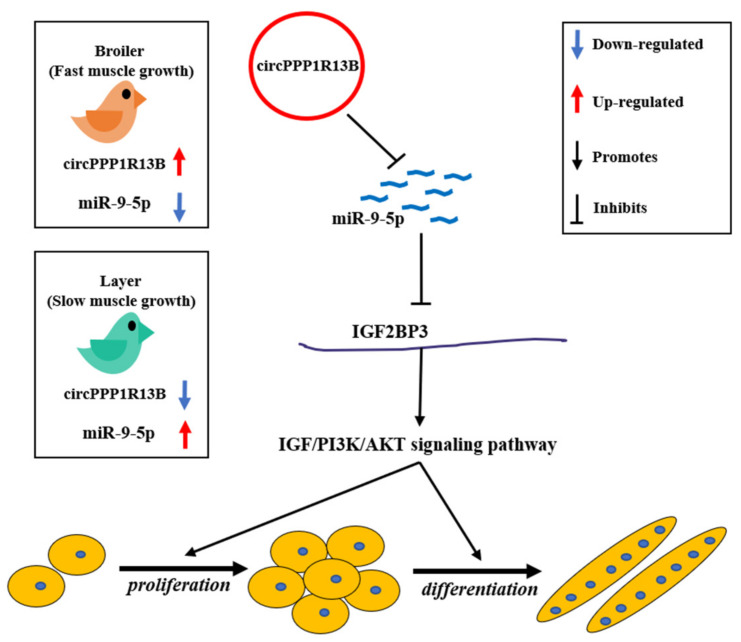
A schematic model of circPPP1R13B regulates chicken SMSC proliferation and differentiation. CircPPP1R13B highly expressed in the breast muscle of FMGB compared to that in SMGL, whereas miR-9-5p highly expressed in the breast muscle of SMGL compared to FMGB. Furthermore, circPPP1R13B promotes chicken SMSC proliferation and differentiation via targets miR-9-5p to active IGF2BP3 mediated IGF/PI3K/AKT signaling pathway.

In conclusion, we found that a novel circRNA circPPP1R13B was highly expressed in the breast muscle of FMGB compared to SMGL, and confirmed that circPPP1R13B could promote SMSC proliferation and differentiation in chicken. Furthermore, we established that circPPP1R13B targets SMGL enriched miR-9-5p to repress the negative effects of miR-9-5p on chicken SMSC proliferation and differentiation. Therefore, the results obtained in this study revealed that circPPP1R13B could act as a potential target factor in chicken molecular breeding to improve skeletal muscle growth in broilers (Figure 6).

## Data Availability

Data will be available upon request to the corresponding author.

## Supplementary Materials

The following are available online at https://www.mdpi.com/article/10.3390/ani11082396/s1, Table S1: RNA oligonucleotides in this article, Table S2: qRT-PCR primers in this article, Table S3: The predicted target miRNAs of circPPP1R13B.
